# First-line antibiotic susceptibility pattern of toxigenic *Corynebacterium diphtheriae* in Indonesia

**DOI:** 10.1186/s12879-019-4675-y

**Published:** 2019-12-11

**Authors:** Dominicus Husada, Sugi Deny Pranoto Soegianto, Indra Suwarin Kurniawati, Adi Pramono Hendrata, Eveline Irawan, Leny Kartina, Dwiyanti Puspitasari, Parwati Setiono Basuki, Ismoedijanto

**Affiliations:** 1 Department of Child Health, Faculty of Medicine Universitas Airlangga/Dr. Soetomo Academic General Hospital, Surabaya, Indonesia; 2 Balai Besar Laboratorium Kesehatan Daerah (BBLK), Surabaya, Indonesia

**Keywords:** First line antibiotics, Penicillin, Erythromycin, Susceptibility pattern, *Corynebacterium diphtheriae*, Toxigenic, Indonesia

## Abstract

**Background:**

Diphtheria has been reported as an outbreak in some regions in Indonesia, most especially in East Java Province. Resistance to penicillin, erythromycin, and other antibiotics, single or multiple, has been reported in several studies. This study aims to evaluate the first-line antibiotic susceptibility pattern of toxigenic *Corynebacterium diphtheriae* isolates.

**Methods:**

This descriptive observational study was performed from August to November 2018. *C. diphtheriae* isolates were collected from diphtheria patients and carriers in East Java from 2012 to 2017 and kept at the Balai Besar Laboratorium Kesehatan Daerah Surabaya or the Public Health Laboratory of Surabaya. Sample selection was done by random cluster sampling. The sensitivity test by E-test®of the five antibiotics (penicillin, oxacillin, erythromycin, azithromycin, and clarithromycin) was done to determine the minimum inhibitory concentration (MIC). The Clinical and Laboratory Standards Institute M45A (2015) *Corynebacterium spp.* for penicillin and erythromycin was used as standard.

**Results:**

From 114 targeted isolates, 108 were viable and toxigenic. The E-test was performed on the viable isolates. The majority of the hosts were male (58.3%), with median (range) age of 6.5 (1–14) years. Half of the samples were from the 1 to 5-year-old age group. The isolates were acquired much more from patients (78.7%) than carriers (21.3%) and from pharyngeal swab (74.1%). Most of these isolates were from Madura Island (47.2%) and the northern and eastern parts of the province (horseshoe area). Mitis isolates were the major variant (76.9%). The susceptibility pattern of *C. diphtheriae* to erythromycin was better than that to penicillin. The E-test result for penicillin was 68.52% susceptible, 31.48% intermediate, and 0% resistant (MIC range, < 0.016 to 2 μg/L) and for erythromycin (MIC range, < 0.016 to > 256 μg/L) was 85.2% susceptible, 12% intermediate, and 2.8% resistant The MIC range for oxacillin was 1 to 96 μg/L, while for both azithromycin and clarithromycin were <  0.016 to > 256 μg/L.

**Conclusion:**

The susceptibility rate of *C. diphtheriae* to erythromycin is higher than that to penicillin. The regular update of antibiotic selection to the national guidelines is recommended. The MIC reference standard to azithromycin and clarithromycin is also needed.

## Background

There has been a high number of diphtheria cases in Indonesia since 2011. East Java Province is the most endemic area of these cases, and the only province officialy declared with diphtheria outbreak. Almost 80% of the cases in Indonesia are in this province [1, 2]. Despite many efforts to tackle the outbreak, these diphtheria problems have not been stopped [2]. All aspects of prevention and treatment, including the antibiotic resistance factor, have been evaluated in this province. Basically, there are two main treatments of diphtheria, anti diphtheria serum (ADS) and antibiotics. ADS binds the free toxin in the circulation. The toxin that is already inside the cell cannot be neutralized by this ADS. Antibiotic therapy plays other significant roles in diphtheria management [3–6]. The availability of ADS in Indonesia becomes very limited due to supply shortage [7, 8]; for several years, many patients in Indonesia could not received ADS. Just recently, the only national vaccine manufacturer in Indonesia has produced ADS to fulfill this gap. This condition might add a more significant role for antibiotics as an important treatment for diphtheria.

Antibiotics are needed to kill the toxigenic *Corynebacterium diphtheriae*, stop the toxin production and the dissemination in symptomatic patients and clinical disease, and reduce the spread from asymptomatic carriers and the colonization of close contacts [3–6]. Penicillin and macrolide are groups of empirical antibiotics used to eradicate toxigenic *C. diphtheriae* based on the World Health Organization (WHO) and the Centers for Disease Control and Prevention (CDC) guidelines for the treatment of diphtheria [9, 10]. Penicillin is the standard treatment for diphtheria since the 1940s [11]. Some studies have already reported on the increase of resistant isolates of this toxigenic bacteria [12–15].

The emergence of antibiotic resistance to *C. diphtheriae* has made a serious concern in some countries [3, 16–20]. It is also possible that the decreased susceptibility to penicillin is a cause of *C. diphtheriae* eradication failure [3, 21]. During this period of high number of diphtheria cases in East Java, there are no data regarding the first-line antibiotic susceptibility pattern of *C. diphtheriae*. The first-line antibiotics include penicillin and macrolides.

Erythromycin has been recommended as the drug of choice for diphtheria for such a long time, but several studies have reported the decrease of susceptibility to this drug [12–15]. The other problem with this drug is its gastrointestinal effect [4]. In our clinical settings, only a few people can finish the 7-day procedure of erythromycin. The cardiac side effect of erythromycin has been reported in the 1980s [22]. However, no such data in diphtheria patients and carriers have been collected and reported in Indonesia. Erythromycin is available in the country in oral forms only.

The WHO has also added azithromycin as part of the standard antibiotics for diphtheria [23]. Azithromycin is successfully used for other infectious diseases such as typhoid fever [24]. Azithromycin has not been used widely for diphtheria patients in Indonesia but it is well-known by many clinicians and is available all over the country. Clarithromycin, also mentioned by a few guidelines [25], is used only by a few clinicians at present. This drug has several advantages. It has milder gastrointestinal effects compared to erythromycin [26], and in Indonesia has lower costs than that of azithromycin.

Aside from all the first-line antibiotics mentioned above, oxacillin, one of the drugs from the penicillinase-resistant-semisynthetic-penicillin group, has been added in this study. This drug has some advantages which include good bioavailability and administration choice by intramuscular or intravenous injection [27]. Its potency, especially for Gram-positive bacteria, and availability in most areas in Indonesia (sometimes better than the availability of penicillin) are also taken into account.

This study aims to evaluate East Java toxigenic *C. diphtheriae* isolates for their susceptibility to penicillin, oxacillin, and macrolide group of antibiotics. The result of this study, hopefully, can be used as a basis to maintain or change the antibiotic choices as one of the main treatments of diphtheria in Indonesia.

## Methods

### Laboratory

Balai Besar Laboratorium Kesehatan Daerah (BBLK) Surabaya or the Public Health Laboratory of Surabaya is one of the two referral laboratories for diphtheria in Indonesia. This laboratory is located in Surabaya, the second-largest city in Indonesia, covering the whole area of the eastern part of the country. The BBLK has international accreditation and is a referral laboratory for diphtheria, polio, and measles cases in Indonesia. All *C. diphtheriae* isolates from East Java and the eastern part of Indonesia were collected at this laboratory.

### Collection of toxigenic *C. diphtheriae* isolates

*C. diphtheriae* isolates were collected from diphtheria patients and carriers during outbreaks in East Java, especially from 2012 to 2017. After that, specimens from each swab (nose and throat) were placed in Amies transport media (Deltalab SL, Barcelona, Spain) and transported within 24 h at 2 °C to 8 °C from the original districts to the BBLK, where the specimens were cultured in Hoyle media (Oxoid, Basingstoke, Hampshire, United Kingdom) for 24 to 48 h. Then, Gram-positive colonies were recultured on Columbia agar (Oxoid). Several following tests using pyrazinamidase, cysteinase, urea, and nitrate [28] and biochemical tests (API CorynE-test Kit; Biomerieux, Marcy l’Etoile, France) were performed. The final procedure was a modified Elek test to confirm the toxigenicity [29]. The whole process took 5 to 7 days. After all thE-tests, these isolates, were later stored in trypticase soy broth with 20% glycerol at –80 °C.

### Sample size and sample selection

The minimal sample size was calculated by the Krejcie and Morgan formula for a limited population with α = 0.05, Z = 1.96, and deviation (d) = 0.05. The total isolate collection from 2011 to 2017 at the BBLK was 200. Based on the formula, the minimal sample requirement was 104.

Among all isolates at the BBLK, the samples were chosen by random cluster sampling system. The cluster was based on the district’s distribution. In every cluster, the isolates were proportionally selected based on the proportion of samples at that particular district to the total isolates. Madura Island, and the northern and eastern parts of the province were prioritized because most of the patients and carriers during the diphtheria outbreak in East Java were from these areas [2]. These heavily affected districts were called collectively as the horseshoe area based on its shape in the map [30]. People in the horse-shoe area were predominantly from Madura ethnicity. This ethnic group has specific characteristics. Madura people generally have lower economic and educational status [30].

### Viability and toxigenicity tests

The viability and toxigenicity tests were performed from August to November 2018. The isolates were recultured on Hoyle media and Columbia agar and then re-identified with somE-tests, similar to the standard procedure during the initial collection process. Also the modified Elek test was performed to check the toxigenicity. If the isolates were viable and toxigenic, then the E-test procedure was done.

### E-test strips

The E-test strips of benzylpenicillin PG256 (code BMX502508), erythromycin EM256 (code BMX510518), azithromycin AZ256 (code BMX501618), clarithromycin CH256 (code BMX508708), and oxacillin OX256 (code BMX520518) were used. All E-tests were from Biomerieux®.

### E-test procedure and interpretation of the results

The E-test® was performed on the viable and toxigenic isolates based on the instructions by the manufacturer. Basically, the isolates were placed on Columbia blood agar plate (BAP) and incubated for 24 h. On the viable isolates, the E-test strips were placed on the inoculation site of BAP using manual applicator forceps. The temperature was set at 35 °C on ambient atmosphere for 16 to 20 h.

This method was used to determine the minimum inhibitory concentration (MIC; in μg/L) of different antimicrobial agents against microorganisms as tested on agar media using overnight incubation. The MIC interpretative standard in this study was based on the Clinical and Laboratory Standards Institute (CLSI) M45A (2015) *Corynebacterium spp.* for penicillin and erythromycin [31]. The MIC standard for penicillin was ≤ 0.12, 0.25 to 2, and ≥ 4 μg/ml for susceptible (S), intermediate (I), and resistant (R), respectively, while the MIC standard for erythromycin is ≤ 0.5, 1, and ≥ 2 μg/ml, for S, I, and R, respectively [31]. There were no MIC criteria for oxacillin, azithromycin and clarithromycin for *Corynebacterium* on CLSI, therefore the MIC data were presented without any interpretation of susceptibility.

In this study, both “intermediate” and “resistant” categories were grouped as “less susceptible”. Multiresistant antibiotics were defined as resistant to more than one drug.

### Statistical analysis

Statistical analysis was performed with a descriptivE-test by IBM® SPSS® version 20 for Mac OS.

## Results

Initially, 114 isolates were selected. Among them, 6 isolates were nonviable; therefore, 108 viable isolates could bE-tested for the E-test at the end. The characteristics of the 108 toxigenic *C. diphtheriae* isolates and the hosts are shown in Table 1. Most of the hosts were patients (78.7%), and boys (58.3%), and belong to the ≤ 5-year-old age group (50%). The predominant districts were Madura Island and the northern and eastern parts of the province as mentioned earlier. Most biotypes of *C. diphtheriae* in this study were mitis (76.9%). Eighty isolates (80 of 108; 74.1%) came from the pharynx. The years with the most cases were 2013 (35.2%) and 2017 (32.4%).

**Table 1:** Characteristics of toxigenic *C. diphtheriae* isolates and the hosts

Host characteristics		
Sex	(n)	(%)
Male	63	58.3
Female	45	41.7
Age, median (min-max)	6.5 (1–14) years	
Group age	(n)	(%)
1–5 years	54	50
> 5–10 years	33	30.6
> 10–15 years	21	19.4
Host status	(n)	(%)
Patients	85	78.7
Carriers	23	21.3
Isolate characteristic		
Year of isolate collection	(n)	(%)
2012	7	6.5
2013	38	35.2
2014	7	6.5
2015	15	13.9
2016	6	5.6
2017	35	32.4
Area of isolate collection	(n)	(%)
Madura Island	51	47.2
Northern and eastern parts of East	35	32.4
Java (Horseshoe area)		
Other parts of East Java	22	20.4
Sites of isolate collection	(n)	(%)
Pharyngeal swab	80	74.1
Nose swab	28	25.9
*C. diphtheriae* variant	(n)	(%)
*Gravis*	25	23.1
*Mitis*	83	76.9
*Intermedius*	0	0
*Belfanti*	0	0

The MIC range of penicillin (< 0.016 to 2 μg/L) and erythromycin (< 0.016 to > 256 μg/L) and the resistance number of isolates are shown in Table 2. The susceptible rate for erythromycin (85.19%) was higher than that of penicillin (68.52%). There were no resistant isolates to penicillin, compared to the three resistant isolates to erythromycin. For both antibiotics, most of the less susceptible isolates were gravis biotype [24 of 36 (66.67%) for penicillin, and 16 of 16 (100%) for erythromycin]. All less susceptible isolates to erythromycin were also less susceptible to penicillin.

**Table 2:** MIC, variants and CLSI interpretation of toxigenic *C. diphtheriae* isolates for penicillin and erythromycin

Antibiotics and biotype	MIC range (μg/L)	Interpretation according to CLSI M45A		
		S	I	R
Penicillin	< 0.016 to 2	74 (68.52%)	36 (31.48%)	0 (0%)
*Gravis*		1	24	0
*Mitis*		73	12	0
Erythromycin	< 0.016 to	92 (85.19%)	13 (12.04%)	3 (2.77%)
*Gravis*	> 256	9	13	3
*Mitis*		83	0	0

Source: CLSI M45A (2015) penicillin for *Corynebacterium spp.* (S) ≤ 0.12 μg/L, (I) 0.25 to 2 μg/L, and (R) ≥4 μg/L; for erythromycin: (S) ≤ 0,5 μg/L, (I) 1 μg/L (R), and ≥ 2 μg/L; S=Susceptible; R = Resistant; I=Intermediate

**Fig. 1:**
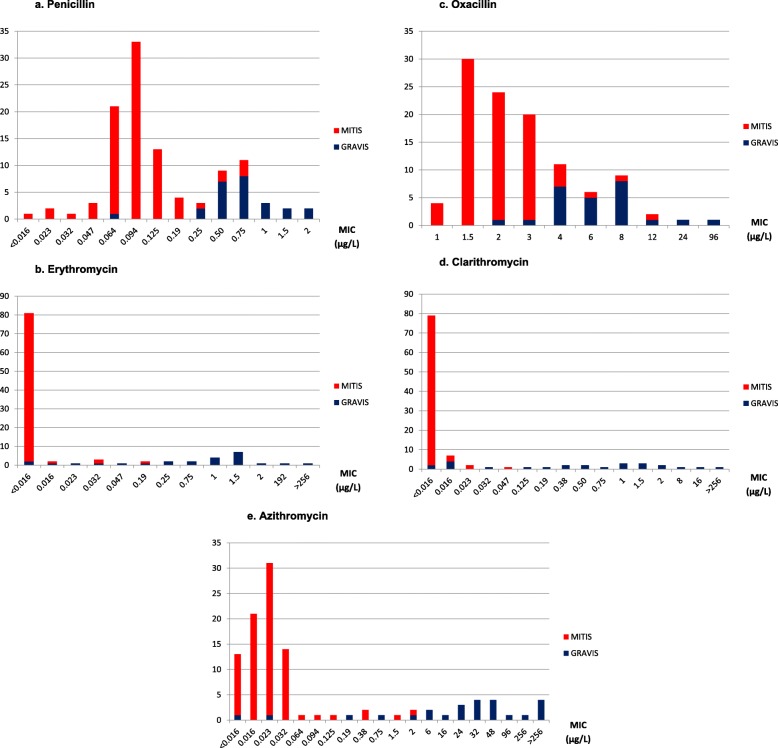
MIC of the five antibiotics. **a** Penicillin; **b** erythromycin; **c** oxacillin; **d** clarithromycin; **e** azithromycin Vertical axis: Number of isolates Horizontal axis: MIC (μg/L) Biotypes: mitis (red); gravis (blue)

**Fig. 2:**
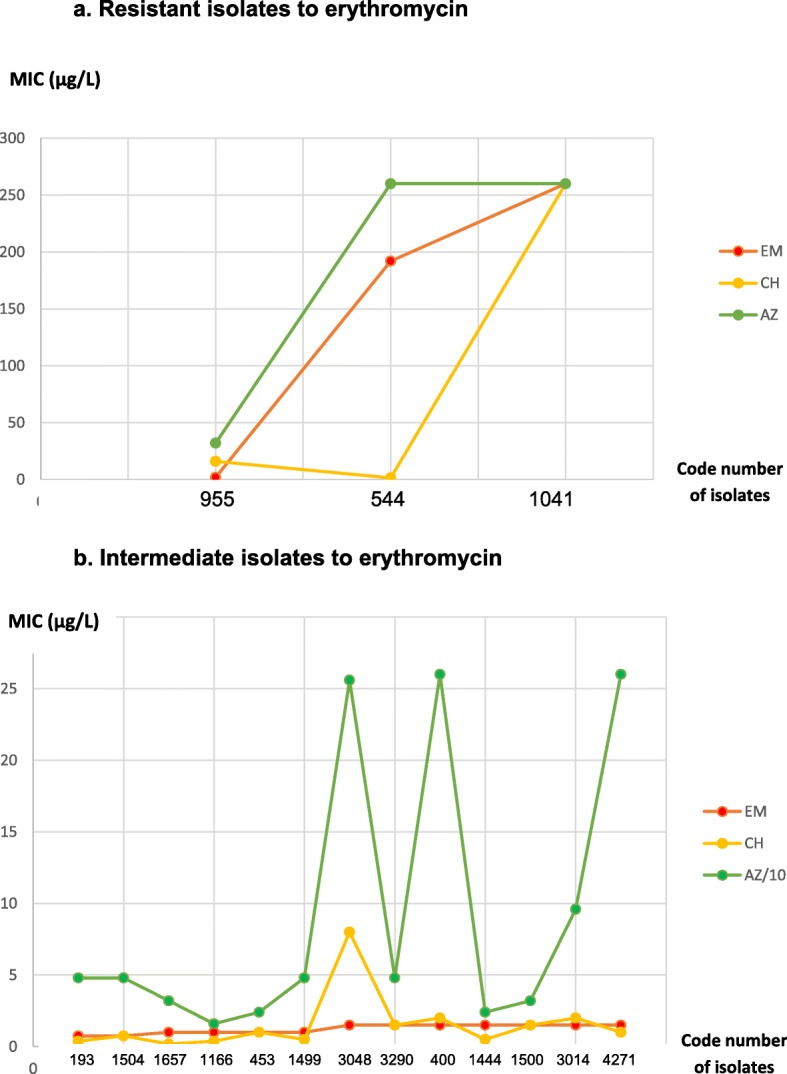
Comparison of MIC of the three macrolides, focusing on less susceptible isolates to erythromycin **a** Resistant isolates to erythromycin (3 isolates). **b** Intermediate isolates to erythromycin (13 isolates) Vertical axis: MIC (μg/L); In Fig. 2b, the MIC of azithromycin was one tenth of the real value Horizontal axis: code number of isolates Color: erythromycin/EM (red), clarithromycin/CH (yellow), azithromycin/AZ (green)

The MIC data of oxacillin, azithromycin, and clarithromycin are shown in Table 3. The MIC range for oxacillin, azithromycin, and clarithromycin were 1 to 96, < 0.016 to > 256, and <  0.016 to > 256 μg/L, respectively. Figure 1a–e shows the bar diagram of the MIC of the five antibiotics. Higher MIC was found for oxacillin. For all antibiotics, most gravis biotypes were in higher MIC than mitis. Figure 2 compares the MIC of three macrolides, focusing on isolates with reduced susceptibility to erythromycin. Most of the less susceptible isolates to erythromycin also had high MIC to azithromycin, but possibly not to clarithromycin.

**Table 3:** MIC of *C. diphtheriae* to Oxacillin, Azithromycin, and Clarithromycin

MIC (μg/L)	OXACILLIN (range, 1 to 96 μg/L)			AZITHROMYCIN (range, < 0.016 to > 256 μg/L)			CLARITHROMYCIN (range, < 0.016 to > 256 μg/L)		
	Total	Mitis	Gravis	Total	Mitis	Gravis	Total	Mitis	Gravis
< 0.016	0	0	0	13	12	1	79	77	2
0.016	0	0	0	21	21	0	7	3	4
0.023	0	0	0	30	30	1	2	2	0
0.032	0	0	0	14	14	0	1	0	1
0.047	0	0	0	0	0	0	1	1	0
0.064	0	0	0	1	1	0	0	0	0
0.094	0	0	0	1	1	0	0	0	0
0.125	0	0	0	1	1	0	1	0	1
0.19	0	0	0	1	0	1	1	0	1
0.25	0	0	0	0	0	0	0	0	0
0.38	0	0	0	2	2	0	2	0	2
0.50	0	0	0	0	0	0	2	0	2
0.75	0	0	0	1	0	1	1	0	1
1	4	4	0	0	0	0	3	0	3
1.5	30	30	0	1	1	0	3	0	3
2	24	23	1	2	1	1	2	0	2
3	20	19	1	0	0	0	0	0	0
4	11	4	7	0	0	0	0	0	0
6	6	1	5	2	0	2	0	0	0
8	9	1	8	0	0	0	1	0	1
12	2	1	1	0	0	0	0	0	0
16	0	0	0	1	0	1	1	0	1
24	1	0	1	3	0	3	0	0	0
32	0	0	0	4	0	4	0	0	0
48	0	0	0	4	0	4	0	0	0
64	0	0	0	0	0	0	0	0	0
96	1	0	1	1	0	1	0	0	0
128	0	0	0	0	0	0	0	0	0
192	0	0	0	0	0	0	0	0	0
256	0	0	0	1	0	1	0	0	0
> 256	0	0	0	4	0	4	1	0	1

## Discussion

In 2017, the WHO has released the latest operational protocol for the clinical management of diphtheria. The recommended antibiotics were penicillin, such as procaine benzylpenicillin and aqueous benzylpenicillin (penicillin G), and macrolides, such as erythromycin and azithromycin [23]. Other recommendations such as from the South African National Institute for Communicable Diseases and United Kingdom (UK) guidelines for the control of diphtheria have stated that clarithromycin as a drug of choice could be used to clear the *C. diphtheriae* infection [25]. In addition, clarithromycin as the main antibiotic for diphtheria have been already used by some clinicians [32, 33]. These antibiotics are active against *C. diphtheriae* [34]*.* In Indonesian Guidelines, the two main antibiotics are procaine penicillin and erythromycin [35]. There is no registered intravenous penicillin or intravenous erythromycin in Indonesia. Based on the WHO recommendations, some clinicians use azithromycin as another choice of antibiotic [23]. As far as we concerned, there is no publication regarding resistance data and the clinical use of azithromycin and clarithromycin for diphtheria patients and carriers in Indonesia.

During the outbreak, the boys slightly outnumbered the girls, which also happened in the distribution of the host of these isolates. In several diphtheria outbreaks in the world, such as in the former Soviet Union, in which most of the patients were adults, females were predominant [36]. The assumption was that males received more vaccination for diphtheria and tetanus because of military duties and being more likely to have infected wounds [37]. In many developing countries, such as India and Vietnam, the majority of the patients and carriers were children [5, 14, 38], while most of the patients during the former Soviet Union outbreak were adults [36]. The high incidence in children ages < 5 years old showed low vaccination coverage. In contrast, the good primary vaccination coverage among babies would create a shift, and most of the patients were preschool and school-aged children [3, 16]. However, historical data showed that the shift to older ages has already been started before the mass immunization era due to several factors such as crowding and poor personal hygiene [16].

Mitis biotypes were predominant in this study, similar to several results from France, [39], Algeria, [40], Malaysia, [41], Brazil, [3], United Kingdom, [15], and previous studies in Indonesia [42, 43]. In Poland and another study in United Kingdom, the majority of the biotypes were gravis [4, 44]. Gravis biotypes in Vietnam and Algeria caused more severe clinical forms [5, 40]. .

Our result in Table 2 showed higher susceptibility of *C. diphtheriae* to erythromycin compared to penicillin. Most less susceptible isolates to penicillin were gravis biotype. Rockhill et al. in 1982 using disk diffusion method (10 IU penicillin and 15 μg erythromycin) showed that all (133 of 133) *C. diphtheriae* isolates from Jakarta were susceptible to penicillin [42]. Kneen et al. in 1998 also using disk diffusion method (10 IU penicillin and 15 μg erythromycin) showed that all (15 of 15) isolates of their study in Vietnam were susceptible to penicillin. Gravis biotypes caused more severe clinical forms [5]. The result of the susceptibility test by Engler et al. in 2001 in the United Kingdom (with 410 *C. diphtheriae* isolates from 1988 to 1998) showed that penicillin G was active against all isolates (MIC_90_, 0.43 mg/L). Agar dilution method and confirmation with E-test were used in this study [4]. In Brazil, Pereira et al. reported that resistance to penicillin G (MIC_90_, 0.19 μg/L) was found in 14.8% (7 of 47) of thE-tested isolates. They also used disk diffusion method and E-test for the study. Since at that time CLSI did not provide the MIC breakpoints obtained by disk diffusion method for coryneform bacteria, the authors used the breakpoints for *Staphylococcus aureus* [3]. In France, a study by Patey et al. found that all isolates (38 of 38) were susceptible to penicillin. This study used disk diffusion method (6 μg penicillin) [39]. In New Delhi India, Sharma et al. reported that all isolates (54 of 54) were susceptible to penicillin. Also, this study used disk diffusion method (10 IU penicillin and 15 μg erythromycin) [45]. More recently, in Canada, Bernard et al. showed 100% susceptibility of 195 *C. diphtheriae* isolates from all over the country to penicillin. This study used broth microdilution and CLSI 2015 as standard [20]. In contrast to all studies above, an epidemiology study of diphtheria and antimicrobial resistance among diphtheria cases in Bijapur District, Karnataka, India, from 2012 to 2015 by Mohankumar et al. showed very high incidence of penicillin resistance, of about 92% (24 of 26) of cases [46]. Unfortunately we could not get any additional data of this study from the literature. Another study from Thailand by Paveenkittiporn et al. also showed reduced susceptibility to penicillin. All isolates (41 of 41) were in the intermediate category against penicillin. Broth microdilution and CLSI 2015 were used as standard [19]. An evaluation of isolates from 1992 to 2005 in Algeria found reduced susceptibility in 90 of 157 (57.3%) samples. This study used E-test with CLSI 2015 as standard [40].

If studies in Brazil and the United Kingdom used the CLSI 2015 standard, the reduced susceptibility rate to penicillin would have been different [3, 4]. With standards from the Brazilian study (CLSI 2006, *S. aureus* to penicillin), our data found resistant isolates to penicillin at least as many as a quarter of the total isolates. If the study by Perreira et al. used CLSI 2015 as standard, the resistance rate would be zero and the reduced susceptibility rate would only be 10%, while if the study by Engler et al. used CLSI 2015, none of the isolates would be categorized as resistant.

In Indonesia, a recent research in 2018 by Sariadji et al. from the Indonesian National Institute of Health Research and Development reported that 10.5% (6 of 57) of *C. diphtheriae* isolates were resistant to penicillin. The disk diffusion method (10 μg penicillin and 15 μg erythromycin) and CLSI 2010 were used in this study. The MIC standard of penicillin in CLSI 2010 is different from CLSI 2015, the newer edition. Most of these isolates were collected from the western side of Indonesia [43]. If our study used the CLSI 2010 as standard, the susceptibility rate to penicillin would be better. Until now, the majority of institutions and guidelines still recommend penicillin as the drug of choice for the treatment of diphtheria. At the same time, some authors hypothesized that penicillin tolerance has been the cause of *C. diphtheriae* infection treatment failure [3, 10, 21, 23].

In this study, MIC range to oxacillin was higher than that to penicillin. The MIC range to oxacillin was 1 to 96 μg/L. The susceptibility evaluation could not be done for this drug since there was no standard in CLSI 2015. In Brazil, a study by Perreira et al. reported that 46 of 47 isolates showed resistance to oxacillin and/or ampicillin [3]. Another case report by Pennie et al. about the misidentification of toxigenic *C. diphtheriae* in a child with endocarditis revealed resistance to oxacillin but susceptibility to other antimicrobial agents such as penicillin G and erythromycin [47]. In France, Patey et al. showed that all isolates (38 of 38) were susceptible to amoxicillin [39]. Some older studies by Weiss et al., Martinez-Martinez et al., and Perreira et al. applied breakpoints of other bacteria, mostly *S. aureus*, to penicillin, oxacillin, and ampicillin to evaluate *C. diphtheriae* [3, 17, 48, 49]. Based on the high MIC level in this study we predicted that oxacillin could not be used for diphtheria.

The WHO Manual for laboratory diagnosis of diphtheria describes that erythromycin is marginally more active than penicillin [9]. Several experts and guidelines consider erythromycin as a drug of choice because it has better in vitro activity than penicillin, such as in the Vietnam study (MIC range, 0.025–0.05 mg/L) [5]. However, in other studies, erythromycin was reported to have high resistance [3–5, 43], while in our study, it was still susceptible in 85.2% of the isolates (92 of 108). Although 12% (13 of 108) of the isolates had decreased susceptibility but the resistance rates were relatively low, only 2.8% (3 of 108) had MIC of 2, 192, and > 256 μg/L. Overall, the susceptibility to erythromycin in this study was better than that to penicillin.

The first report of erythromycin-resistant strain was in 1973 by Jellard and Lipinski [50]. Farfour et al. in 2011 evaluated 42 invasive *C. diphtheriae* isolates. All isolates were susceptible to erythromycin, but six of them had reduced susceptibility to penicillin G (MIC range, 0.38–0.5 mg/L) [51]. If Farfour et al. used CLSI 2015 as standard, none of the isolates would be categorized as resistant to penicillin. Pereira et al. also reported that most *C. diphtheriae* isolates were sensitive to erythromycin (MIC_90_, 0.75 μg/mL), and only 4.2% (2 of 47) of the isolates showed decreased susceptibility (MIC, 1.0 μg/mL) [3]. Engler et al. in the United Kingdom found only 5 of 410 isolates with reduced susceptibility to erythromycin and other macrolides. These isolates came from Australia and Vietnam [4]. A study in Thailand found high susceptibility to erythromycin [19]. A similar reported was found in Algeria, in which 100% (157 of 157) were susceptible to erythromycin [40]. This result was similar to a study in Delhi, India (54 of 54 isolates) [45]. Patey et al., in France found 2 of 38 isolates with reduced susceptibility to erythromycin [39]. In Indonesia, Rockhill et al. reported the susceptibility of all isolates to erythromycin (133 of 133); recently, Sariadji et al. reported that only 5.3% (3 of 57) of the isolates were resistant to erythromycin [42, 43]. Other studies in other countries showed resistance of *C. diphtheriae* to erythromycin [5, 39, 52]. In Vietnam, 27% (4 of 15) of isolates showed decreased susceptibility to erythromycin. The author also reported slower fever clearance and higher gastrointestinal side effects associated with this drug [5]. Bernard et al., in Canada showed reduced susceptibility of 33 of 195 (16.9%) *C. diphtheriae* isolates to erythromycin [20].

Zasada recorded more publications of erythromycin-resistant *C. diphtheriae* compared to penicillin-resistant isolates [17]. Resistance to erythromycin could occur in the sub-inhibition level of that antibiotic. Some investigators found the plasmid-mediated resistance pattern [5, 53]. Others described the gene acquisition related resistant mechanism, such as the *ermX* (erythromycin methylase enzyme class X) gene in *Corynebacterium spp* [5, 17]. Barraud et al. found that *C. diphtheriae* can harbor integrons. This genetic feature would give the isolates the capability to easily acquire new gene cassettes, such as *erm,* which encode resistance to erythromycin [54].

This study could not evaluate the susceptibility to azithromycin and clarithromycin due to the absence of the accepted breakpoints for C*orynebacterium* in CLSI 2015. We considered that the MIC data for azithromycin and clarithromycin did not show much difference from the MIC data for erythromycin (all macrolides had the same MIC range). Our data showed higher MIC for gravis biotype, as also found in penicillin. Engler et al. in the United Kingdom found that all isolates with reduced susceptibility to erythromycin also showed reduced susceptibility to azithromycin and clarithromycin. Based on the MIC, the authors stated that the clarithromycin was the most active among other macrolides (MIC_90_ for clarithromycin, erythromycin, roxithromycin, and azithromycin was 0.008, 0.026, 0.03, and 0.058 μg/L, respectively) [4]. According to Wilson, the advantages of clarithromycin are as follows: it has fewer side effects and could be given twice daily. Azithromycin can be given once daily with also fewer gastrointestinal side effects than erythromycin. These three macrolides might have a similar activity against *C. diphtheriae* in vitro [55]. In Pereira et al. study, azithromycin (MIC_90_, 0.064 μg/mL) was active against *C. diphtheriae* [3]. Patey et al in 1997 showed a similar activity between erythromycin and azithromycin [56].

Our results showed that all isolates with reduced susceptibility to erythromycin had high MIC to azithromycin and partly also to clarithromycin. These isolates also had reduced susceptibility to penicillin. Once again, all isolates with resistance or lower susceptibility were gravis. To our knowledge, in the literature, the studies about the resistance pattern to azithromycin and clarithromycin were limited, most probably because the MIC standard for both macrolides was not available.

As there were no resistant isolates to penicillin in this study, we could not evaluate the multiresistant *C. diphtheriae*. These multiresistant isolates were reported in some other studies, with various definitions. Perreira et al. in Brazil showed antimicrobial multiresistance patterns of 95.74% (45 of 47) *C. diphtheriae* strains to 4 to 7 antibiotics. Only 2 of 47 isolates did not show the multiresistance state [3]. In Vietnam, 3 of 15 (20%) isolates were multiresistant to penicillin and erythromycin [5]. A previous study in Indonesia by Sariadji et al found 12% multiresistant isolates, which included 4 of 57 isolates that were resistant to 3 to 5 antibiotics [43]. In Canada, at least 18 multiresistant isolates were reported by Bernard et al [20]. The first case of multiresistant *C. diphtheriae* in Canada was resistant to erythromycin (MIC, 2 μg/L) and some other antibiotics, but not penicillin. Broth microdilution was used in this study [18]. However, data from Russia (2.3%), India (0%), and Poland (0%) showed very limited multiresistant strains [45, 57, 58].

The main implication of this study would affect the antibiotic choice in Indonesia. At present, penicillin was more recommended than erythromycin. Our result showed that the erythromycin was better than penicillin.

Antibiotic resistance variability, in terms of time of incidence, and types of antibiotic, was found not only in Indonesia [42, 43], but also all over the world [3–5, 19, 20, 40, 41, 58]. Good quality and exact recommendation would not be possible without regular susceptibility testing [19, 20, 40, 43]. Although at this moment our country was not able to do this recommendation, it should be put as one of the priorities.

This study used the E-test based on practicability and easiness, but it is not the standard method according to the CLSI. However, many published literatures used E-test, [3, 4, 40, 59, 60] and the disk diffusion method, [3, 5, 39, 43] and, for more recent studies, standard broth microdilution methods [19, 20, 61]. Several literatures found high correlation of the E-test and the disk diffusion method [3, 4, 17, 48, 49, 59].

The other limitation of this study was we did not examine the deoxyribonucleic acid (DNA) sequence of *C. diphtheriae* to analyze the association between the genetic aspect and the MIC. Genetic information is suggested to be done regularly [62]. Further study may be focused in this issue.

## Conclusions

The susceptibility rate of toxigenic *C. diphtheriae* isolates to erythromycin is better than that to penicillin. The updated version of antibiotic selection and choice in the national guidelines for diphtheria is recommended. This may include the willingness to provide erythromycin injection, which is not available in Indonesia at this moment. Moreover, many variations of antibiotic resistance from many parts of the world, and minimal data from our country lead to the need for regular susceptibility testing. The international MIC reference standard for other macrolide groups, especially azithromycin and clarithromycin is also needed.

## Data Availability

The raw dataset of MIC is included as supplementary material.

## Abbreviations

ADS: Antidiphtheria serum
BAP: Blood agar plate
BBLK: Balai besar laboratorium kesehatan
CDC: Center for disease control and prevention
CLSI: The clinical and laboratory standard institute
DNA: Deoxyribonucleic acid
I: Intermediate
MIC: Minimum inhibitory concentration
R: Resistant
S: Susceptible
UK: The United Kingdom
WHO: World Health Organization
